# WHAT MATTERS TO OLDER SURGICAL PATIENTS AND THEIR CARERS DURING THEIR HOSPITALIZATION?

**DOI:** 10.1093/geroni/igac059.2040

**Published:** 2022-12-20

**Authors:** Janani Thillainadesan, Helen Box, Leanne Kearney, Vasi Naganathan, Michelle Cunich, Sarah Aitken, Sue Monaro

**Affiliations:** Department of Geriatric Medicine and Centre for Education and Research on Ageing (CERA), Concord Hospital, Concord, New South Wales, Australia; Concord Hospital, Concord, New South Wales, Australia; Concord Hospital, Concord, New South Wales, Australia; Concord Hospital, Concord, New South Wales, Australia; University of Sydney, Sydney, New South Wales, Australia; Concord Hospital, Concord, New South Wales, Australia; Concord Hospital, Concord, New South Wales, Australia

## Abstract

Older adults account for a growing proportion of the surgical population. We investigated patient- and carer-reported experiences of hospitalization in this group. A mixed-methods study using telephone and postal surveys of recently hospitalized vascular surgical patients aged ≥ 65 years at an acute care academic hospital. Qualitative data were thematically analyzed using a framework approach. In total 46 patients (mean age 77 years, 78% male) and nine carers were surveyed. Nine (20%) patients were frail (Clinical Frailty Scale score >4). The majority of patients reported overall they felt their views were listened to (n=42, 89%), were kept informed (n=39, 83%) and were asked about their pain level (n=37, 79%). Among the nine carers, seven reported overall they felt their views were listened to (n=42, 89%) and were kept informed (n=39, 83%). Thematic analysis of responses to open-ended survey questions about their experience of hospitalization revealed four key themes: receiving fundamental care including hygiene and nutrition, importance of the hospital environment such as sleep and meals, being informed and involved in health care decision-making, and treating pain and deconditioning to help recovery. Findings from this study identified that older adults admitted to hospital for surgical care, and their carers, highly value care that meets fundamental needs and involves them in decision-making and recovery. These priorities can be addressed through Age-Friendly Health System initiatives such as the implementation of the “4Ms” of geriatrics care: what matters, medication, mobility and mentation.

